# Psychometric characteristics of the health care empowerment questionnaire in a sample of patients with arthritis and rheumatic conditions

**DOI:** 10.1111/hex.13196

**Published:** 2021-01-27

**Authors:** Erin Knight, Kathleen Carluzzo, Karen E. Schifferdecker, Emily Creek, Rebecca L Butcher, Guy S. Eakin

**Affiliations:** ^1^ Dartmouth College Geisel School of Medicine Hanover NH USA; ^2^ The Dartmouth Institute for Health Policy and Clinical Practice Center for Program Design and Evaluation Lebanon NH USA; ^3^ Arthritis Foundation Atlanta GA USA

**Keywords:** empowerment, health care empowerment questionnaire, patient participation, patient‐reported experience measures, psychometrics, validation study

## Abstract

**Background:**

Patient empowerment can improve health‐related outcomes and is important in chronic conditions, such as arthritis. This study aimed to validate the Health Care Empowerment Questionnaire (HCEQ), a patient‐reported experience measure of empowerment, for use with patients with arthritis and other rheumatic diseases.

**Methods:**

The HCEQ measures Patient Information Seeking (or Involvement in Decisions) and Healthcare Interaction Results (or Involvement in Interactions) and asks respondents to answer questions in two ways: whether they feel something happened and its importance to them. Face validity was assessed through qualitative data (n = 8, nominal group technique; n = 55, focus groups). Measure structure was assessed through confirmatory factor analysis (CFA); internal consistency was also assessed (n = 9226). Test‐retest reliability was assessed with sub‐sample of participants (n = 182).

**Results:**

We found adequate face validity of the HCEQ for patients with arthritis. The CFA indicated good fit to the data for the two‐factor structure of the HCEQ (RMSEA = 0.075; CFI = 0.987; TLI = 0.978; SRMR = 0.026). Internal consistency was strong (α=0.94 for both subscales). Test‐retest reliability was moderate for Patient Information Seeking (ICC=0.67) and good for Healthcare Interaction Results (ICC=0.77).

**Conclusions:**

The HCEQ, with modifications, demonstrated promising psychometric properties within this sample, laying the foundation for further assessment. This work supports the HCEQ as an appropriate instrument for examining experiences with and perceived importance of empowerment in individuals with arthritis and other rheumatic conditions.

**Patient Contribution:**

Patients contributed to the assessment of face validity. As a measure of patient empowerment, the HCEQ’s use can enable further participation of patients in health care.

## 1 INTRODUCTION

Patient‐reported outcome measures (PROMs) are valuable in increasing patient engagement in clinical care by tracking self‐reported outcomes and informing treatment decisions.^1^ Patient‐reported experience measures (PREMs) fulfil a similar purpose to PROMs, but they are different in that they measure what happened while care was provided to a patient, and the patient's perspective of their experience,^2^ rather than health status or disease progression.

One aspect of patient experience with health care is patient empowerment, defined by the World Health Organization as ‘a process through which people gain greater control over decisions and actions affecting their health’. ^3^ This concept focuses on an individual's own behaviours and beliefs in interactions with their health‐care teams, rather than the influence of the health‐care team and provider on a patient's experience. Studies support that patient empowerment, such as increased patient involvement in medical decisions, can lead to improved health outcomes, quality of life and satisfaction with health care.^4^, ^5^, ^6^, ^7^

### 1.1 Health care empowerment questionnaire

There are a number of measures that purport to assess the multidimensional concept of patient empowerment. One instrument, the Health Care Empowerment Questionnaire (HCEQ), was developed with a sample of ageing adults in Canada.^8^ The HCEQ measures three aspects of patient empowerment that had been proposed in the literature: degree of control, or consideration of who is involved in making decisions^9^; involvement in interactions, or the ability and opportunity to communicate needs and initiate requests with a health‐care provider^10^; and involvement in decisions, or actively obtaining the information necessary to make rational decisions.^11^ A concept analysis conducted more recently defines patient empowerment in similar terms, defining key attributes as the ability to effect change based on personal behaviour and the social environment, self‐determination and ability for autonomy, and a process for obtaining self‐management tools that enables empowerment.^12^

With its original population of adults aged 75 and older in Canada, the HCEQ demonstrated good test‐retest and internal consistency reliability on its three scales.^8^ Specific health issues in the original sample were not provided, though participants were included if they were expected to experience a functional decline. Its measure structure was tested through an exploratory factor analysis (EFA) and validated with a confirmatory factor analysis (CFA), providing evidence for a three‐factor structure and support for construct validity in their sample. A Persian version of the HCEQ has been validated with a sample of reproductive age women in Iran,^13^ but validation studies of the English version with specific populations, beyond healthy adults, have not been published.

### 1.2 Patient empowerment in arthritis, a chronic condition

In 2018, the authors (EK, KES and RLB) collaborated with the Arthritis Foundation (including authors EC and GSE) to develop the Live Yes! INSIGHTS survey, which uses PROMs and PREMs to longitudinally track member experiences.^14^ The INSIGHTS survey is administered quarterly, and results are used to guide regional and national programming, resources, advocacy and research as part of the Arthritis Foundation's larger Live Yes! Arthritis Network,^15^ its patient‐facilitated network to support an estimated 20 million individuals with arthritis.

In developing the Live Yes! INSIGHTS programme, the Arthritis Foundation was interested in measuring patient empowerment to understand the experiences of patients with different characteristics (eg types of arthritis, race/ethnicity, socio‐economic status) and from different geographic regions. By understanding needs and potential differences related to patient empowerment, the Foundation could then tailor their programming and patient education, which may enhance patients’ empowerment in their care and in decision making.^16^

Patient empowerment is particularly important in populations of patients with chronic conditions, such as arthritis, as these patients interact frequently with the health‐care system and therefore have more opportunity for interactions with their providers. Arthritis and other rheumatic conditions are one of the leading causes of chronic pain in the United States^17^ and rank seventh in the 2010 National Hospital Discharge Survey as the first‐listed diagnosis on the hospital discharge.^18^ In the National Ambulatory Medical Survey, osteoarthritis alone accounted for 11 147 000 primary care visits.^19^ Similarly, in a 13‐year longitudinal study, patients with rheumatoid arthritis were significantly more likely than controls to utilize services provided by general or specialty care physicians (OR = 1.75), particularly early in the disease.^20^ Ostensibly, more frequent interactions with the health‐care system indicate that patient empowerment is a critical construct to understand within populations with chronic conditions such as arthritis, particularly when evidence has shown that patient empowerment can lead to better outcomes.^4^, ^7^ However, the importance patients place on patient empowerment, including information seeking and involvement in decision making, depends on the patient and the decision being made.^21^, ^22^

### 1.3 Validation of the HCEQ with a population of patients with arthritis

Given the importance of patient empowerment, patient input was used to develop the Live Yes! INSIGHTS programme, which resulted in the selection of the HCEQ (see Schifferdecker et al for more information).^14^ The purpose of this study was to assess the psychometric properties of the HCEQ in a sample of patients with arthritis. This is critical as this is the first study to evaluate the HCEQ with a sample like this in the United States.

## 2 METHODS

We conducted our psychometric validation study in two phases. In phase 1, we assessed face validity using qualitative methods. In phase 2, we used data from completed INSIGHTS surveys to assess measure structure, internal consistency and test‐retest reliability. Both phases of the study were deemed not human subjects research by our Institutional Review Board.

### 2.1 Phase 1: Qualitative data to assess face validity

Details of the qualitative study are described in detail elsewhere.^14^ Briefly, we used a modified Delphi and virtual nominal group technique (NGT; n = 8), and then six focus groups (n = 55), to get input on the empowerment measure to use in INSIGHTS. Participants primarily included adults with arthritis, though a health‐care provider and measurement expert also participated. Participants in the NGT were asked to rate three patient empowerment or self‐advocacy measures, one of which was the HCEQ, selected through a critical literature review emphasizing psychometric quality.^23^ Ratings were summarized and used to facilitate discussion of the measures among NGT participants, including whether the measures adequately captured patient empowerment or self‐advocacy. After the HCEQ was selected, information about how likely patients would be to complete the survey at multiple points over time was gathered from the focus groups. We recorded and obtained transcripts for both the NGT and the focus groups and assessed face validity through descriptive ranking data collected during the NGTs, along with a content analysis of the NGT discussion and focus group transcripts.

### 2.2 Phase 2: Survey deployment and quantitative analyses

#### 2.2.1 Study design and participants

For the quantitative psychometric analyses in the current study, we used cross‐sectional data from the Arthritis Foundation's Live Yes! INSIGHTS programme (‘INSIGHTS’),^14^ in which patients in the United States with arthritis are invited to complete patient‐reported outcome and experience measures periodically online in a non‐clinical setting. This survey was finalized based on the findings of phase 1.

Surveys were administered through an online platform (Qualtrics™, Provo, UT). There were two ways in which participants could provide data: through an anonymous URL provided on the Arthritis Foundation website or through an email invitation using contact lists from people who had previously participated in Arthritis Foundation programming. Participants were encouraged to take the survey as often as they saw fit, and some of these administrations occurred within the seven‐ to 14‐day window that was determined to be sufficient for assessing test‐retest reliability, based on previous research with other patient experience measures.^24^, ^25^, ^26^, ^27^ The first survey completed by each eligible participant was used for this study (or two survey administrations occurring seven to 14 days apart for test‐retest reliability).

To confirm measure structure and assess internal consistency, we extracted a sample of 9226 individuals from the INSIGHTS population. Participants’ data were included in this study if they were at least 18 years old, English speaking, had completed a survey between 19 March 2019 and 15 March 2020 (due to the potential impact of COVID‐19 stay‐at‐home orders), provided some demographic information to ensure they were a unique participant, self‐reported having a diagnosis of one or more types of arthritis, reported having seen a doctor within the last six months (a requirement for completing the HCEQ) and provided complete responses on key variables assessed in this study.

We used a subset of this sample who completed the survey twice within seven to 14 days to assess test‐retest reliability (n = 182). In addition to the general inclusion criteria listed above, all participants used in the test‐retest analysis were included only if they had not had surgery, a hospital visit or medication change between survey administrations, to ensure similar patient status at each administration.

#### 2.2.2 Measure

We administered the HCEQ^8^ along with basic demographic questions. The HCEQ includes ten questions that fall under three factors, which the HCEQ authors labelled Degree of Control, Involvement in Decisions (which we call Patient Information Seeking) and Involvement in Interactions (which we call Healthcare Interaction Results). In phase 1 of the study, the NGT participants, mostly patients themselves, recommended removing the Degree of Control scale as it was not perceived to be relevant or important to patients (see face validity results for more information). Therefore, we only included seven questions from the two other factors. Additionally, because we were testing this questionnaire with a sample of patients with arthritis and other rheumatic conditions, we modified the instructions slightly to read, ‘Consider the health services you have received *for your arthritis* during the last 6 months and keep this in mind as you answer the questions below. These questions will first assess your feelings, and then the importance you give to different situations related to these services *for your arthritis’*.

Participants answered questions using a Likert scale response from one to four. Respondents were asked to answer each of the seven questions in two ways: with regard to their experience (‘did you feel that…’) and their perceptions of the importance of the item (‘how important is it that…’). As per the original article, we created scale scores by obtaining the cross‐product of the Feelings and Importance responses for each of the items and summing these items within the Patient Information Seeking and Healthcare Interaction Results scales. See Table 1 for details about the survey and its items. In addition to the HCEQ and demographic items, the full survey included the Patient‐Reported Outcomes Measurement Information System (PROMIS) PROMIS‐29 Profile v2.1 (29 items) and PROMIS Emotional Support Short Form v2.0 (four items). These measures are not included in the present analyses.

**TABLE 1:** Original HCEQ scales and items (Degree of Control scale not used)

	Feelings responses prompt	Importance responses prompt	Items
Patient Information Seeking (Involvement in Decisions)	During the last 6 months, did you feel that…	During the last 6 months, how important is it that…	you asked for explanations (Info 1) you asked questions (Info 2) you asked for advice (Info 3)
Healthcare Interaction Results (Involvement in Interactions)	During the last 6 months, did you feel that…	During the last 6 months, how important is it that…	you were able to talk to a professional to answer your questions (Result 1) your choices were respected (Result 2) you obtained all the information you wanted (Result 3) you got the help you needed (Result 4)
*Degree of Control* *(not used)*	During the last 6 months, did you feel that…	During the last 6 months, how important is it that…	that you and your loved ones decide the need for the health care and services that you and your loved ones decide the type of health care and services that you and your loved ones decide the amount of health care and services

Abbreviation: HCEQ, Health Care Empowerment Questionnaire.

#### 2.2.3 Data analysis

To assess measure structure (and construct validity), we ran a confirmatory factor analysis (CFA), applying the same measurement structure to this sample of adults with arthritis and other rheumatic conditions, as was applied in the original HCEQ psychometric development.^8^ A CFA was used since the aim of this analysis was to confirm an existing factor structure presented in previous research^8^ with a new population of patients with arthritis and other rheumatic diseases.^28^, ^29^

We examined two of three factors presented in the original model: Involvement in Decisions (Patient Information Seeking) and Involvement in Interactions (Healthcare Interaction Results). In addition, we examined an alternative model separating out the Feelings and Importance responses, rather than using the cross‐products, for two reasons: 1) to determine whether other models demonstrated better fit to the data and 2) because previous literature using patient empowerment measures included items similar to the HCEQ Feelings responses and examined the impact of importance separately.^21^, ^22^ Because patients may feel differently about the importance of patient empowerment,^21^, ^22^ it could be valuable to look at this separately from their perspectives on what occurred in their interactions with health‐care providers, allowing these to be used as separate scales speaking to related constructs.

To assess internal consistency reliability, we calculated Cronbach's alphas for each of the two scales, Patient Information Seeking and Healthcare Interaction Results. We also examined whether an improvement would be made if any items were removed from either scale. To assess test‐retest reliability, we examined intraclass correlations (ICCs) between the scale scores at Time 1 and Time 2 for both scales. While there is some debate over what should be considered adequate test‐retest reliability, we considered an ICC of above 0.7 to be adequate under present circumstances.^30^ These analyses were also run on the two additional models using the Feelings and Importance responses separately.

## 3 RESULTS

Demographic information for each sample is presented in Table 2.

**TABLE 2:** Demographic data for each sample: qualitative data sample, full quantitative sample and test‐retest sub‐sample (of the full quantitative sample)

		Qualitative sample (face validity)		Full sample		Test‐retest sub‐sample	
		N	Statistic	N	Statistic	N	Statistic
Race/ ethnicity	White, non‐Hispanic	35	52.2%	7716	79.3%	155	85.2%
	Black or African American	22	32.8%	479	4.9%	6	3.3%
	Hispanic or Latino	1	1.5%	445	4.6%	8	4.4%
	Asian	‐	‐	93	1.0%	2	1.1%
	American Indian or Alaska Native	1	1.5%	41	0.4%	1	0.5%
	Middle Eastern or North African	‐	‐	14	0.1%	‐	‐
	Native Hawaiian or Pacific Islander	‐	‐	11	0.1%	‐	‐
	More than one race	‐	‐	149	1.5%	3	1.6%
	No response	8	11.9%	786	8.1%	7	3.8%
Gender	Male	7	10.4%	1124	11.6%	13	7.1%
	Female	56	83.6%	8547	88.2%	169	92.9%
	Other	‐	‐	19	0.2%	‐	‐
	No Response	4	6.0%	‐	‐	‐	‐
Education	Less than high school	1	1.5%	85	0.9%	‐	‐
	High school diploma/GED	5	7.5%	915	10.1%	21	11.5%
	Some college	19	28.4%	3174	35.2%	62	34.1%
	4‐year college degree	14	20.9%	2523	28.0%	49	26.9%
	Graduate degree	14	20.9%	2324	25.8%	50	27.5%
	No response	14	20.9%	‐	‐	‐	‐
Age	18‐44 years	9	13.4%	1201	12.8%	20	11.0%
	45‐64 years	26	38.8%	4175	44.5%	92	50.5%
	65 + years	29	43.3%	4006	42.7%	70	38.5%
	No response	3	4.5%	‐	‐	‐	‐
Avg. Age	Average age (SD)	56	61.7 (14.5)	9382	60.3 (13.9)	182	60.2 (12.4)
Work status^a^	Employed full time/part‐time	N/A	‐	3249	36.1%	58	31.9%
	Unemployed	N/A	‐	257	2.9%	8	4.4%
	Unable to work	N/A	‐	1371	15.2%	32	17.6%
	Other (retired, student, volunteer, etc)	N/A	‐	4119	45.8%	83	45.6%
	No response	N/A	‐	‐	‐	1	0.5%

Note

Avg = average; SD = standard deviation.

^a^ Note: Work status was not asked of participants in the NGT and focus groups.

### 3.1 Face validity

After discussion during the virtual NGT in phase 1, participants selected the HCEQ as their preferred measure of patient empowerment or self‐advocacy, pointing to its ability to ‘provide much more valuable information’ as compared to other surveys, lending support to its face validity. Overall, on their initial ratings of the HCEQ and its items, more than half of participants reported that they thought the HCEQ was moderately to extremely useful.

When examining ratings across the three subscales, participants indicated that one HCEQ subscale (‘Degree of Control’) was not as easy to answer. In discussing this during the group, participants reported that items such as ‘you and your loved ones can decide the amount of healthcare’ and ‘you and your loved ones decided the need for healthcare and services’ were confusing or not applicable because they would not expect to have control in those circumstances due to their insurance. Because participants felt that the Degree of Control scale was difficult to answer and not as relevant, we decided to remove it to further increase the HCEQ’s face validity and assess the measure structure of the scale in this form.

In focus groups, when reviewing the modified HCEQ (without the Degree of Control scale), participants reported that they would be willing and interested in completing the measure as often as monthly for tracking, indicating to some extent that they found the measure to be a valid assessment of relevant constructs. They reported that people would be more interested in completing the survey if they understood the purpose and benefits of completing it; a few participants also reported that they would be more likely to complete the measure if they knew it was a valid tool, and not ‘just something [that the Foundation] cooked up’, emphasizing the importance of further validation of the scale.

### 3.2 Construct validity and measure structure

Due to the results of the NGT, we removed the third factor in the original model (Degree of Control). The items that loaded onto the two remaining scales through an EFA and confirmed with a CFA in the original article were specified in the same way in the current model. Thus, a two‐factor model was fit, with three items on Patient Information Seeking and four items on Healthcare Interaction Results.

Missingness was low (n = 508, 5.21%), and thus, cases with missing values were deleted listwise. Since the assumption of multivariate normality was not met for both the Patient Information Seeking and Healthcare Interaction Results scales, model fit was examined using maximum‐likelihood (ML) estimation with the Satorra‐Bentler adjustment, which adjusts for non‐normal data. Model fit indices are provided in Table 3, alongside the results from the original development and validation article. Table 3 also includes information on the models tested, that is the number of factors and their names, and which items were specified to load onto each factor to show any differences between each model.

**TABLE 3:** CFA model fit indices, internal consistency (Cronbach's alpha) and test‐retest reliability results (Spearman's rank correlation coefficients) from the original HCEQ validation article, and for the present combined model and present Feelings/Importance models

		Original HCEQ validation from Gagnon et al (with 3 factors)^8^	Combined model^a^	Feelings only model	Importance only model
CFA model fit statistics (n = 9226)^b^	Chi‐square (*df*)	68.23 (31), *P*< .001	685.229 (13), *P*< .001	571.809, *P*< .001	438.883, *P*< .001
	RMSEA (90% CI)	0.052	0.075 (0.070 ‐ 0.080)	0.067 (0.062 ‐ 0.072)	0.059 (0.054 ‐ 0.064)
	CFI	0.979	0.987	0.985	0.984
	TLI	‐	0.978	0.979	0.974
	SRMR	‐	0.026	0.026	0.028
Internal consistency (n = 9410‐9681) ^b^	Patient Info. Seeking	α=0.79	α=0.94	α=0.91	α=0.92
	Healthcare Interaction Results	α=0.79	α=0.94	α=0.93	α=0.89
Test‐retest reliability (n = 182) ^b^	Patient Info. Seeking	ICC=0.62	ICC=0.67*	ICC=0.62*	ICC=0.57*
	Healthcare Interaction Results	ICC=0.60	ICC=0.77*	ICC=0.78*	ICC=0.59*
Model tested: factors (and items specified to load on each factor)		1. Patient Information Seeking (Info 1: F x I, Info 2: F x I, Info 3: F x I) 2. Healthcare Interaction Results (Result 1: F x I, Result 2: F x I, Result 3: F x I, Result 4: F x I) 3. Degree of Control (Control 1: F x I , Control 2: F x I, Control 3: F x I)	1. Patient Information Seeking (Info 1: F x I, Info 2: F x I, Info 3: F x I) 2. Healthcare Interaction Results (Result 1: F x I, Result 2: F x I, Result 3: F x I, Result 4: F x I)	1. Patient Information Seeking (Info 1: F, Info 2: F, Info 3: F) 2. Healthcare Interaction Results (Result 1: F, Result 2: F, Result 3: F, Result 4: F)	1. Patient Information Seeking (Info 1: I, Info 2: I, Info 3: I) 2. Healthcare Interaction Results (Result 1: I, Result 2: I, Result 3: I, Result 4: I)

Abbreviations: CFA, confirmatory factor analysis; CFI, Comparative Fit Index; CI, confidence interval; *df*, degrees of freedom; F, Feelings responses; HCEQ, Health Care Empowerment Questionnaire; I, Importance responses; ICC, intraclass correlation coefficient; ML, maximum likelihood; RMSEA, root mean square error of approximation; SRMR, standardized root mean square residual; TLI, Tucker‐Lewis Index.

^a^ The present models all used maximum‐likelihood estimation with Satorra‐Bentler correction. For verification, this model was also estimated using asymptotic distribution‐free (ADF) estimation, which makes no assumption of normality, and the results were comparable.

^b^ All of the Ns provided are for the present sample (not for the original validation article).

* *P *< .001 (for present sample).

**FIGURE 1:**
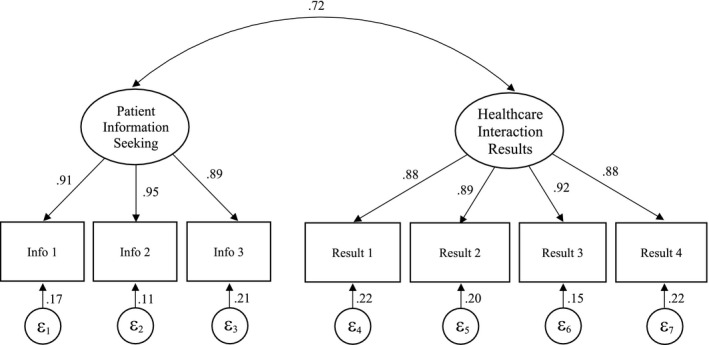
Final two‐factor model and factor loadings for the Health Care Empowerment Questionnaire (HCEQ), with standardized coefficients

The model (n = 9226) indicated good overall fit to the data with regard to most model fit indices, RMSEA =0.075 (90% CI =0.070‐0.080), CFI =0.987, TLI =0.978 and SRMR =0.026. Chi‐square for the model was significant (χ^2^(13) =685.229, *P* < .001), which is not unexpected since chi‐square is sensitive to large samples (ie over 200). The model and factor loadings are provided in Figure 1.

**FIGURE 2:**
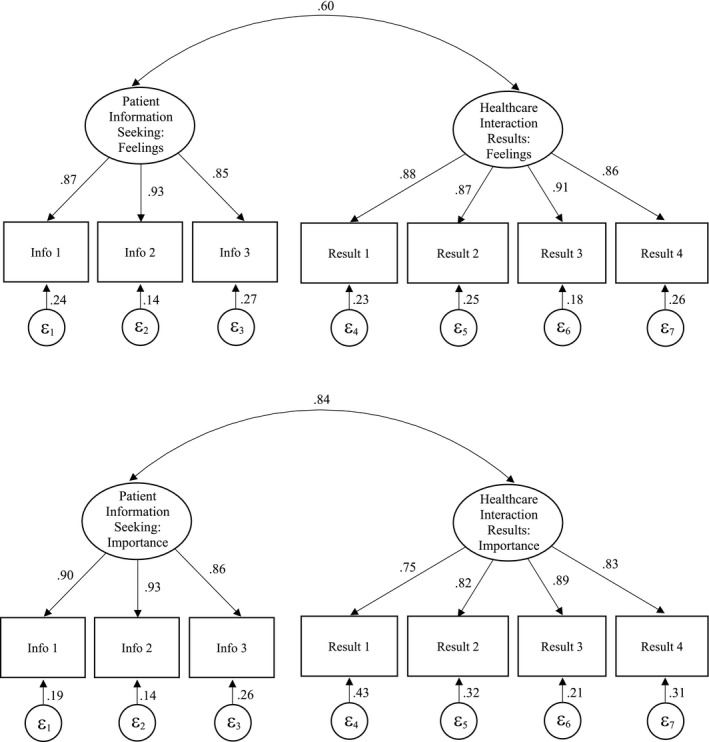
Separate Feelings and Importance Health Care Empowerment Questionnaire (HCEQ) models and factor loadings, with standardized coefficients

Another set of models was run to determine whether separating out the Feelings and Importance responses demonstrated better fit to the data (see Table 3 for more information on items included). Two separate models were specified: one with the Feelings responses and one with the Importance responses, both using the two scales: Patient Information Seeking (three items) and Healthcare Interaction Results (four items). Scale scores were obtained by summing the individual items in each scale, rather than summing the cross‐products. Using ML estimation with the Satorra‐Bentler adjustment, the model for Feelings (n = 9226) indicated good overall fit to the data, RMSEA = 0.067 (90% CI =0.062‐0.072), CFI = 0.985, TLI = 0.979 and SRMR = 0.026, again with the exception of the chi‐square, χ^2^ (13) =571.809, *P* < .001. The same was true for Importance (n = 9226), showing good overall fit to the data, RMSEA =0.059 (90% CI =0.054‐0.064), CFI =0.984, TLI = 0.974 and SRMR =0.028, with the exception of the chi‐square, χ^2^ (13) =438.883, *P* < .001. The models and factor loadings are provided in Figure 2. While these separate models did indicate good fit, they were not a substantive improvement over the original combined model.

### 3.3 Internal consistency reliability

Cronbach's alphas were run, and missing data were deleted listwise as missingness was low. For the combined model using cross‐products, Cronbach's alpha for Patient Information Seeking (α = 0.94) and Healthcare Interaction Results (α = 0.94) were good (Table 3).

When the responses were separated, Cronbach's alphas for the Feelings responses were somewhat lower, but acceptable, for Patient Information Seeking (α = 0.91) and Healthcare Interaction Results (α = 0.93). Cronbach's alphas for the Importance responses were still within the acceptable range for Patient Information Seeking (α = 0.92) but were slightly lower than 0.9 for Healthcare Interaction Results (α = 0.89).

### 3.4 Test‐retest reliability

We used ICCs to assess test‐retest reliability using 182 eligible cases (Table 3). Test‐retest reliability for Patient Information Seeking was low, but adequate (ICC =0.67), and good for Healthcare Interaction Results (ICC= 0.77).

When the responses were separated, test‐retest reliability for the two groups of Feelings responses followed the same trend as the combined model: adequate for Patient Information Seeking (ICC =0.62), but good for Healthcare Interaction Results (ICC =0.78). Test‐retest reliability for the two groups of Importance responses was low for both Patient Information Seeking (ICC =0.57) and Healthcare Interaction Results (ICC =0.59).

## 4 DISCUSSION

While multiple patient‐reported experience measures have been developed and validated with different populations, this is the first study to validate the HCEQ, a measure specifically capturing patient empowerment, with a sample of adults with arthritis and other rheumatic conditions in the United States. This is valuable as patients with arthritis frequently interact with the health‐care system,^18^, ^19^, ^20^ and the ability to assess patient empowerment using a reliable and valid tool could identify opportunities for increasing patient empowerment, and thus potentially related health outcomes.^4^, ^7^

Our study provides preliminary support for the use of the HCEQ in two regards: the data show that it is valid when used with patients with arthritis and other rheumatic conditions and that it displays good psychometric quality compared with the original, despite changes (ie removal of the Degree of Control scale, instructions noting that patients were to respond about health‐care services ‘*for their arthritis’*). We removed the Degree of Control scale due to patient feedback, and also due to the context of the health insurance system in the United States, as the scale implies that patients are always able to make choices about specific medications, for example.

Our sample also had a lower mean age suggesting that the HCEQ is relevant for younger individuals as well, as seen in the original validation study.^8^ We validated the HCEQ’s original measure structure and demonstrated good fit to the data. Additionally, the scales corresponding to each of the two factors, Patient Information Seeking and Healthcare Interaction Results, demonstrated good internal consistency and adequate to good test‐retest reliability.

While the results are promising, there is room for improvement. Test‐retest reliability was lower for Patient Information Seeking (ICC =0.67), though this value was comparable to the intraclass correlation coefficients reported in the original article, which ranged from 0.60 to 0.70.^8^ Because respondents are not asked to reference a specific type of provider, it is possible that patients considered different types of providers or health‐care services when completing the HCEQ at each of the two time points.

Our results also provide preliminary support for the use of the Feelings responses on the HCEQ alone, but not for the Importance responses alone. We found lower test‐retest reliability coefficients using only the Importance responses on Patient Information Seeking and Healthcare Interaction Results, while the Feelings responses produced results that were more comparable to the overall combined model.

### 4.1 Suggested modifications to the HCEQ

Given the above considerations, while the HCEQ in its current form (with our modifications included) displays good psychometric quality for individuals with arthritis, additional modifications could be made for further improvement. For instance, asking about patient empowerment generally over the last 6 months provides an opportunity to look at an array of patient‐provider interactions, but specifying the type of provider may be beneficial for increasing consistency in measurement (ie test‐retest reliability). For instance, a question or prompt could ask patients to think about a specific provider such as their rheumatologist. It is possible that patients exhibit different information‐seeking behaviours with different physicians, or in making different decisions,^21^ and physicians may have varied responses to patient empowerment and self‐advocacy.

Considering the two answer types (Feelings and Importance) separately may also be a beneficial use of the HCEQ. Though the CFA assessing the original model (using the cross‐products of Feelings and Importance responses) produced good fit to the data, so did separate models for the Feelings and Importance responses. If used separately, relationships, or the lack thereof, could be explored between a patient's experiences (Feelings) and the importance they attribute to having that experience (Importance). Indeed, some research suggests that patients’ interest in involvement in health decisions may vary on a number of factors including the type of decision,^21^, ^22^, ^31^ so it could be helpful to examine the importance attributed separately.

### 4.2 Limitations and strengths

Though efforts were made to address issues with the data, some limitations are worth noting. First, we used a sample of convenience that was gathered through the efforts of a national foundation. The demographics of this sample indicate that it is not entirely representative of patients with arthritis and rheumatic conditions in the United States; thus, generalizability should be considered. However, these methods allowed us to recruit over 9,000 participants, which is a strength of the study.

Additionally, we relied upon self‐report of arthritis diagnosis, which we were not able to verify. While confirmation of diagnoses is preferable, a recent meta‐analysis suggested that self‐reported arthritis type (specifically osteoarthritis and rheumatoid arthritis) was sufficiently accurate for large‐scale studies when diagnosis cannot be confirmed.^32^ Also, this was a non‐incentivized study, so there is no perceived incentive to lie. Further, though we were not able to verify diagnoses, the fact that we include participants with different types of arthritis and rheumatic conditions allows us to speak to the use of this measure across these diagnoses. We were also not able to speak to whether these findings relate to a specific type of provider, as we did not ask participants to report the type of provider.

While we did have an adequate sample of patients who completed the HCEQ twice within seven to 14 days, these data were not collected intentionally for the purpose of assessing test‐retest reliability. We aimed to address this as much as possible by ensuring that patients had not changed medications or visited a hospital in between administrations of the HCEQ.

### 4.3 Future directions and conclusions

This study provides initial support for the use of the HCEQ with populations with arthritis and other rheumatic conditions. These findings support the use of the HCEQ in the Arthritis Foundation's Live Yes! Network as an assessment for the construct of patient empowerment with this population. Based on these findings, future directions and next steps may include further validation of the HCEQ with this population. For instance, it may be beneficial to assess additional psychometric qualities, such as concurrent and predictive validity. In doing so, it may also be beneficial to assess whether suggested changes improve the test‐retest reliability of the HCEQ with this population, particularly for the separated Feelings and Importance responses. If so, this would allow for an examination of the relationship between the Feelings and Importance responses.

## CONFLICT OF INTEREST

There are no conflicts of interest to report for any authors.

## AUTHOR CONTRIBUTIONS

Knight participated in the conception and design process, prepared for data acquisition, developed the analysis plan, led the analysis and interpretation of the data, drafted the manuscript and led the revision process, and provided final approval of the version to be submitted. Carluzzo participated in the conception and design process, supported the development of the analysis plan, prepared the data for analysis, supported the interpretation of results, and provided critical revisions to the manuscript and final approval of the version to be submitted. Schifferdecker participated in the conception and design process, prepared for data acquisition, supported the development of the analysis plan, supported the interpretation of results, and provided critical revisions to the manuscript and final approval of the version to be submitted. Creek participated in the conception and design process, prepared for data acquisition, and provided critical revisions to the manuscript and final approval of the version to be submitted. Butcher participated in the conception and design process, prepared for data acquisition, supported the development of the analysis plan, and provided critical revisions to the manuscript and final approval of the version to be submitted. Eakin participated in the conception and design process, prepared for data acquisition, and provided critical revisions to the manuscript and final approval of the version to be submitted.

## Data Availability

Research data are not shared.
